# Assessing the capture of sociodemographic information in electronic medical records to inform clinical decision making

**DOI:** 10.1371/journal.pone.0317599

**Published:** 2025-01-17

**Authors:** Rawan Abulibdeh, Karen Tu, Debra A. Butt, Anthony Train, Noah Crampton, Ervin Sejdić

**Affiliations:** 1 Department of Electrical and Computer Engineering, University of Toronto, Toronto, Ontario, Canada; 2 Department of Family and Community Medicine, University of Toronto, Toronto, Ontario, Canada; 3 North York General Hospital, Toronto, Ontario, Canada; 4 Toronto Western Hospital Family Health Team, University Health Network, Toronto, Ontario, Canada; 5 Department of Family and Community Medicine, Scarborough Health Network, Scarborough, Ontario, Canada; 6 Department of Family Medicine, Queen’s University, Kingston, Ontario, Canada; Ege University, Faculty of Medicine, TÜRKIYE

## Abstract

There is a growing need to document sociodemographic factors in electronic medical records to produce representative cohorts for medical research and to perform focused research for potentially vulnerable populations. The objective of this work was to assess the content of family physicians’ electronic medical records and characterize the quality of the documentation of sociodemographic characteristics. Descriptive statistics were reported for each sociodemographic characteristic. The association between the completeness rates of the sociodemographic data and the various clinics, electronic medical record vendors, and physician characteristics was analyzed. Supervised machine learning models were used to determine the absence or presence of each characteristic for all adult patients over the age of 18 in the database. Documentation of marital status (51.0%) and occupation (47.2%) were significantly higher compared to the rest of the variables. Race (1.4%), sexual orientation (2.5%), and gender identity (0.8%) had the lowest documentation rates with a 97.5% missingness rate or higher. The correlation analysis for vendor type demonstrated that there was significant variation in the availability of marital and occupation information between vendors (*χ*^2^ > 6.0, *P* < 0.05). Variability in documentation between clinics indicated that the majority of characteristics exhibited high variation in completeness rates with the highest variation for occupation (median: 47.2, interquartile range: 60.6%) and marital status (median: 45.6, interquartile: 59.7%). Finally, physician sex, years since a physician graduated, and whether a physician was a foreign vs a Canadian medical graduate were significantly associated with documentation rates of place of birth, citizenship status, occupation, and education in the electronic medical records. Our findings suggest a crucial need to implement better documentation strategies for sociodemographic information in the healthcare setting. To improve completeness rates, healthcare systems should monitor, encourage, enforce, or incentivize sociodemographic data collection standards.

## 1 Introduction

As electronic medical records (EMRs) become more widely used, population-scale real-world clinical data becomes more accessible for biomedical research [1–3]. In 2021, approximately 86% of Canadian family physicians adopted EMRs to some extent [4]. EMRs are electronic systems used in clinical care and healthcare administration to store medical information about individual patients [2, 5]. They contain numerous patient-level variables in structured and unstructured forms [6, 7]. EMRs not only allow for easier capture of data but also allow evaluation of care at the practice population level [6, 8, 9]. This requires summary information about the practice population and individual patients. However, providing summary data about the practice population was not initially considered in their development, and obtaining data from EMRs can be challenging. Generally, EMRs do not have capabilities outside the most basic queries.

The digitization of clinical records provided a new opportunity to integrate sociodemographic information into EMRs for enhancing care at the practice population level [6]. Previous research has shown the complexity of disease risk in which genetics or molecules alone are not enough to model it but rather genetic, social, and environmental factors such as socioeconomic status, race, ethnic background, age, and geolocation all play a role in disease risk throughout population groups [10]. As individuals are positioned in a social status hierarchy from birth, this can affect their overall access to healthcare and disease morbidity and mortality [11]. For example, individuals with higher income, education, and superior occupation have better health and a greater life expectancy [12]. Furthermore, factors such as immigration, race/ethnicity, and globalization have a significant influence on individuals’ health status. Studies on migrants have shown the type of diseases, behaviors, and risk factors diverge in migrant populations when compared to populations in their country of origin [13, 14]. Other social and behavioral factors have been shown to influence health outcomes. A vast body of evidence suggests that sexual minority groups are disproportionately influenced by health issues corresponding to stigma and discrimination [15, 16]. There is a significant need for the collection of sexual orientation information in a clinical setting to better understand and address the root cause of sexual orientation-based discrepancies, improve research in providing more enhanced patient-centered care, and provide suitable patient risk assessment [16–18]. In terms of gender identity, it is quite evident that due to discrimination, transgender individuals face adverse health issues such as chronic physical and mental health conditions when compared to cisgender’s. This includes higher rates of asthma, diabetes, chronic obstructive pulmonary disease, and HIV [19]. The impact of marital status is also a widely studied area in health. Marital status has been linked to mortality risk [20], obesity [21], and chronic conditions that limit social activity [22].

Studying the effects of such factors on health enables the contextualization of patient care to attain more sustainable and equitable health outcomes. Collecting sociodemographic characteristics, can help track and examine disparities in health and healthcare, assess these characteristics as potential confounders, and analyze any association to certain diseases [23, 24]. However, there are various limitations to extracting usable information from EMRs including input variability, lack of coding certain data, and missing or poorly represented data [2, 3, 5, 8, 16, 23, 25]. To realize the potential of research employing EMR data, it is essential to extract high-quality, research-grade information from these clinical data sources [1]. The initial step involves assessing the quality and completeness of the data obtained from EMRs to ascertain its utility for research purposes.

A previous study in Canada using EMR data found that the majority of sociodemographic factors were missing, varied considerably, or were suspected to contain data errors [26]. Two studies characterizing occupation data within EMRs free-text clinical notes found that the use of acronyms/abbreviations, misspellings, ambiguous information, and multiple entries resulted in quality issues in the information that was found [27, 28]. Another study characterized the quality of race and ethnicity data in cancer registries and EMRs of five sources [23]. They found that race data varied significantly based on source with the complete agreement of data across sources being only 39.2% of patients. Previous quality assessment studies on EMR data provide a limited and broad view of the subject with no focus on a wide range of variables that may affect documentation rates. Furthermore, very few studies assess sociodemographic characteristics in EMRs and even less so use Canadian EMR records. According to a systematic review of EMR data quality, only 1 of 37 articles contained Canadian data [3, 29]. More recently, a review on data quality of EMRs involved 35 studies: none of which had Canadian data [3, 30].

While it is known, or at least suspected, that documentation of sociodemographic information is poor in EMR records, an exact quantification of the completeness of the data has yet to be performed. Improvements and measurements of improvements in documentation cannot be made without quantification of the current baseline. Therefore, we set out to determine the rates of documentation of sociodemographic characteristics in the EMRs of family physicians practicing in Ontario, Canada.

## 2 Material and methods

### 2.1 Data source

We used the University of Toronto Practice-Based Research Network (UTOPIAN) Data Safe Haven, which is a repository of de-identified EMR data on over 400 family physicians, 96 clinics, and ∼400,000 patients in Ontario [31]. The three EMR vendors from which the UTOPIAN database involved represent the top three EMR vendors in Ontario and are commonly used in family physician practices in Ontario. Each patient represented in UTOPIAN is uniquely associated with a single provider, and each provider uses only one EMR vendor. This structure eliminates the potential for multilevel interactions due to patients being seen by multiple providers or providers using different EMR vendors.

The social history, risk factors, and health conditions sections in the EMR include information on the sociodemographic characteristics of patients. They were used to assess the completeness of the information in the patient’s medical record. These sections solely represent semi-structured fields and make up the summary information contained in the cumulative patient profile section of the EMR record. The system records the history of patient data, with each entry marked by a timestamp. However, this process occasionally resulted in repetitive entries for the same patient. To minimize redundancy, during the preprocessing stage, we retained only the most recent entry when multiple entries began with identical text. Therefore, the most up-to-date status of each sociodemographic characteristic studied in this project was used in all our analyses.

### 2.2 Patient sample

The cohort of patients for our completeness assessment included adults 18 years of age as of December 31^st^, 2021 since certain characteristics such as marital status and occupation were much less likely to be documented in children and youth. Then, we grouped entries by eligible patients and merged the data from the semi-structured fields of the lifestyle, risk factors, social, and medical history fields of the cumulative patient profile portion of the EMR record. No unstructured or fully structured fields were included in the analysis. To obtain a proper representation of the UTOPIAN database, we randomly sampled 1.5% of patients from each clinic. This left us with a cohort of 4,375 patients. We compared the random sample for age, sex, and EMR start date against all patients in the repository to confirm that the sample was representative of patients in the database. Furthermore, we confirmed that the sample included all physicians within each clinic and that the proportion of patients for each physician was reflective of their practice size in the database.

### 2.3 Coding sociodemographic characteristics

To perform the quality assessment, a reference standard was developed by an annotator (RA) manually annotating the social phrases in the cohort based on annotation guidelines. Two labels were created for each characteristic. The first was the documented information found in the semi-structured fields. The second label provided information on the documentation status of each sociodemographic characteristic. Approximately 5% of the sample (219 phrases) was double annotated by the same annotator to reach an intra-rater reliability kappa value of 0.98 for the documentation status labels and 0.96 for the documented information label averaged across all characteristics.

### 2.4 Statistical analyses

#### Correlation analysis of primary care variables on documentation rates

The association between the completeness rates of the sociodemographic data and the various clinics, EMR vendors, and physician characteristics was examined to characterize the underlying effects of such variables on documentation rates. Chi-square tests were used to assess the variation of documentation for each characteristic across vendor types. The variability of documentation rates between various clinics was examined using heatmaps, median, and the first and third quartiles of completeness rates for each variable. Generalized linear mixed models were used to examine the relationship between the documentation rates of the sociodemographic variables and various physicians and clinic characteristics. Generalized linear mixed models were used as they account for the hierarchical structure of the data where patients are nested within providers and providers are nested within clinics. Further potential clustering may be attributed to the nesting of clinics within EMR vendors. However, the variance attributed to this inclusion was minimal and did not produce statistically significant results (p-values ranged from 0.16-0.40). Consequently, this level was excluded from the final analysis.

The variance components for the provider and clinic levels were estimated across all the sociodemographic outcomes (excluding race, sexual orientation, and gender identity as the data was extremely imbalanced resulting in the model not being able to converge), ranging from 0.7 to 1.5 for providers and 0.7 to 2.5 for clinics, with corresponding p-values all < 0.0001. The intraclass correlation coefficients (ICC) were calculated for each outcome to quantify the proportion of variance attributable to differences at the provider and clinic levels. The provider-level ICCs ranged from 0.127 to 0.238, with a median ICC of 0.182. The clinic-level ICCs were higher, ranging from 0.239 to 0.391, with a median ICC of 0.314. On average, the provider-level ICC was 0.185, while the clinic-level ICC was 0.305. These results indicate that a substantial portion of the variance in the outcomes is explained by clustering at the provider and clinic levels, underscoring the importance of accounting for these hierarchical structures in the analysis.

Physician characteristics included the location of practice (rural vs urban), roster size (the number of patients registered to a physician), years since graduation, physician sex, and foreign vs Canadian medical graduate. Clinic characteristics included group size. Group size represents the number of physicians working in the same group (family health team or organization). Some groups are in the same physical office or can be several offices of two or three physicians who work together in the same group. Therefore, we anticipated that any policy or procedure change by physicians/clinics in the types of data and the way it was collected would apply to all physicians within that group. The dependent variable was a binary indicator representing the presence or absence of each sociodemographic variable being analyzed. The independent variables are listed above. To account for the hierarchical structure and correlated outcomes, we included random intercepts for patients nested within providers and providers nested within clinics. All analyses were conducted using the PROC GLIMMIX procedure in SAS Version 9.4 [32].

### 2.5 Supervised machine learning models

Two supervised machine learning models (logistic regression and random forest classifier) were implemented to identify the presence or absence of each characteristic across all adult patients in UTOPIAN. This allowed us to assess whether a similar distribution in quantity could be found in the database by training the models on the reference standard. Furthermore, the development of such tools can provide a reference standard for future studies as to whether machine learning models can adequately predict EMR completeness rates for various factors.

The models were trained on features generated using term frequency-inverse document frequency on the preprocessed text data. Hyperparameter tuning using Bayesian optimization was performed to optimize model performance for each characteristic. We evaluated the models using stratified 10-fold cross-validation to assess model performance and account for class imbalance. The primary evaluation metrics used were precision, recall, and F1-score. Furthermore, Cohen’s kappa coefficient was calculated on a held-out test set of 20% of the patient sample to assess general agreement between the actual label and the predicted label of the best-performing model. All metrics used account for class imbalance since they attenuate by imbalanced distributions [33]. The two machine learning models were evaluated against a simple rule-based algorithm using regular expression searches to derive sociodemographic characteristics. The keyword search terms were determined by primary care physicians and those familiar with primary care EMR data.

## 3 Results

The UTOPIAN database consisted of 381,659 patients provided by 3 EMR vendors (A, B, and C). From those EMR vendors, there were 96 clinics and 408 physicians in total. Table 1 summarizes the information on the EMR vendors included in our study. Nearly 45% of the patients were provided by vendor C, which had 41 clinics and 223 physicians. The patients sampled contain 56.6% females and 43.4% males. If any sociodemographic information was documented, about 84.9% of the information was obtained from the social history section alone, while 14.6% of the information was obtained from a combination of the lifestyle, risk factors, social, and medical history sections.

**Table 1:** Summary of vendor information demonstrating the number of clinics, physicians, and patients per vendor.

Vendors	Clinics	Physicians	Number of Patients (%)
*Vendor A*	45	134	146,195 (38.3%)
*Vendor B*	10	51	64,518 (16.9%)
*Vendor C*	41	223	170,946 (44.8%)
**Total**	96	408	381,659

### 3.1 Patient demographics

Table 2 presents the sociodemographic information obtained from the patient sample. Males and females had a general agreement over the most common category for each sociodemographic factor.

**Table 2:** Summary statistics for each sociodemographic characteristic from the patient sample used in our study. The data are sectioned by male and female demographics.

Characteristic	Female	Male	Characteristic	Female	Male
	N = 2,475 (56.6%)	N = 1,900 (43.4%)		N = 2,475 (56.6%)	N = 1,900 (43.4%)
	(n, (%))	(n, (%))		(n, (%))	(n, (%))
**Place of Birth**			**Gender Identity**		
Canada	118 (4.8)	83 (4.4)	Trans-woman	N/A	<5
Selected Country	132 (5.3)	100 (5.3)	Trans-man	<5	N/A
Other	148 (6.0)	104 (5.5)	Cis-woman	16 (0.6)	N/A
Absent	2,077 (83.9)	1,613 (84.9)	Cis-man	N/A	13 (0.7)
			Non-binary	<5	<5
			Other	0 (0.0)	0 (0.0)
			Absent	2,456 (99.2)	1,885 (99.2)
**Race/Ethnicity**			**Marital Status**		
Asian	5 (0.2)	6 (0.3)	Married	774 (31.3)	649 (34.2)
Black	<5	0 (0.0)	Common-law	32 (1.3)	25 (1.3)
Indigenous	<5	0 (0.0)	Relationship	109 (4.4)	80 (4.2)
Latin	<5	<5	Separated	43 (1.7)	36 (1.9)
Middle Eastern	<5	<5	Divorced	83 (3.4)	31 (1.6)
White	16 (0.6)	16 (0.8)	Widowed	78 (3.2)	25 (1.3)
Mixed Heritage	<5	<5	Single	143 (5.8)	126 (6.6)
Other	0 (0.0)	0 (0.0)	Absent	1,213 (49.0)	928 (48.8)
Absent	2,442 (98.7)	1,871 (98.5)			
**Citizenship Status**			**Occupation**		
Citizen	124 (5.0)	88 (4.6)	Employed	192 (7.8)	154 (8.1)
Permanent resident	<5	<5	Unemployed	56 (2.3)	33 (1.7)
Refugee claimant	0 (0.0)	<5	Retired	187 (7.6)	138 (7.3)
Temporary visa	<5	0 (0.0)	Student	47 (1.9)	16 (0.8)
Immigrant	193 (7.8)	146 (7.7)	Sick leave/Disability	13 (0.5)	12 (0.6)
Other	0 (0.0)	0 (0.0)	Occupation details	665 (26.9)	555 (29.2)
Absent	2,152 (86.9)	1,662 (87.5)	Absent	1,315 (53.1)	992 (52.2)
**Sexual Orientation**			**Education**		
Heterosexual	30 (1.2)	34 (1.8)	Below highschool	6 (0.2)	9 (0.5)
Homosexual	10 (0.4)	28 (1.5)	Highschool	24 (1.0)	28 (1.5)
Bisexual	5 (0.2)	0 (0.0)	Apprenticeship	<5	<5
Not defined	0 (0.0)	0 (0.0)	College	50 (2.0)	27 (1.4)
Asexual	0 (0.0)	0 (0.0)	Diploma/Certificate	<5	<5
Absent	2,430 (98.1)	1,838 (96.7)	University-undergraduate	116 (4.7)	73 (3.8)
			University-graduate	54 (2.2)	29 (1.5)
			Absent	2,221 (89.7)	1,730 (91.1)

Most of the fields for sociodemographic characteristics had limited information except for marital status (51.0%) and occupation (47.2%). The majority of the patients that had any documentation of the characteristics were married (61.3% females, 66.8% males). Entries that contained occupational information in the tables did not detail the employment status; instead, they provided information regarding the patients’ job titles or specializations. However, if employment status was provided the majority of the patients specified they were employed (16.6% females, 17.0% males). Most patients were born outside Canada (70.4% females, 71.1% males) where documentation of citizenship status was rare. The most commonly attained degree was an undergraduate degree (45.7% females, 42.9% males). The most commonly recorded sexual orientation was heterosexual (66.7% females, 54.8% males) followed by homosexual (22.2% females, 45.2% males) with a cis-gender identity (84.2% females, 86.7% males). The most common race of patients was of White descent (48.5% females, 55.2% males).

### 3.2 Machine learning results

Table 3 summarizes the performance metrics across the two machine learning models from the stratified 10-fold cross-validation for individual characteristics and the rule-based baseline algorithm evaluated across the full reference standard. Cohen’s kappa coefficient was calculated on the predictions of the best-performing machine learning model and the baseline algorithm for each characteristic.

**Table 3:** Evaluation metric values of the logistic regression model (LR), random forest classifier (RF), and the rule-based algorithm (RB) for each of the sociodemographic characteristics. The best-performing algorithm is indicated in bold for each metric.

Characteristic	Precision (%)			Recall (%)			F1-score (%)			Cohen’s Kappa		
	LR	RF	RB	LR	RF	RB	LR	RF	RB	ML	RB
**Place of Birth**	88.1	90.2	**93.3**	74.7	**77.2**	34.7	80.7	**83.1**	50.6	**0.79**	0.46
**Race**	80.0	**84.6**	28.3	52.9	**60.7**	49.2	60.7	**66.8**	35.9	**0.72**	0.35
**Citizenship Status**	88.4	**90.4**	27.2	75.0	**78.0**	32.4	81.1	**83.6**	29.6	**0.78**	0.15
**Sexual Orientation**	76.9	79.5	**88.2**	55.5	53.6	**74.5**	63.5	62.6	**80.8**	0.70	**0.80**
**Gender Identity**	**95.0**	94.2	30.1	91.7	85.8	**96.9**	**92.6**	88.9	45.9	**0.87**	0.80
**Marital Status**	95.9	94.9	**97.3**	**95.9**	94.8	56.1	93.4	**94.0**	71.2	**0.86**	0.54
**Occupation**	90.2	82.5	**95.2**	**91.8**	83.5	72.7	**87.0**	84.8	82.4	**0.75**	0.70
**Education**	**85.6**	80.3	15.4	69.7	78.5	**86.5**	76.5	**79.1**	26.1	**0.73**	0.12

Using F1-score as the primary evaluation metric, the random forest classifier showed the best performance for place of birth, race, citizenship status, marital status, and education. The logistic regression model had the best performance for gender identity and occupation. Averaged across all characteristics, the F1-scores were 80.4% and 79.4%, for the random forest classifier and the logistic regression model respectively. The predicted label for all adult patients in the database was obtained based on the best-performing model for each characteristic.

The rule-based algorithm was able to outperform both machine learning models for sexual orientation. This is likely a result of the lack of documentation of sexual orientation in the training sample which produced insufficient data for proper model training. Race and gender identity additionally had a low number of positive cases but contained significant semantic and linguistic variation in the clinical text which cannot be adequately captured by a simple regular expression search.

### 3.3 Data availability

**Fig 1:**
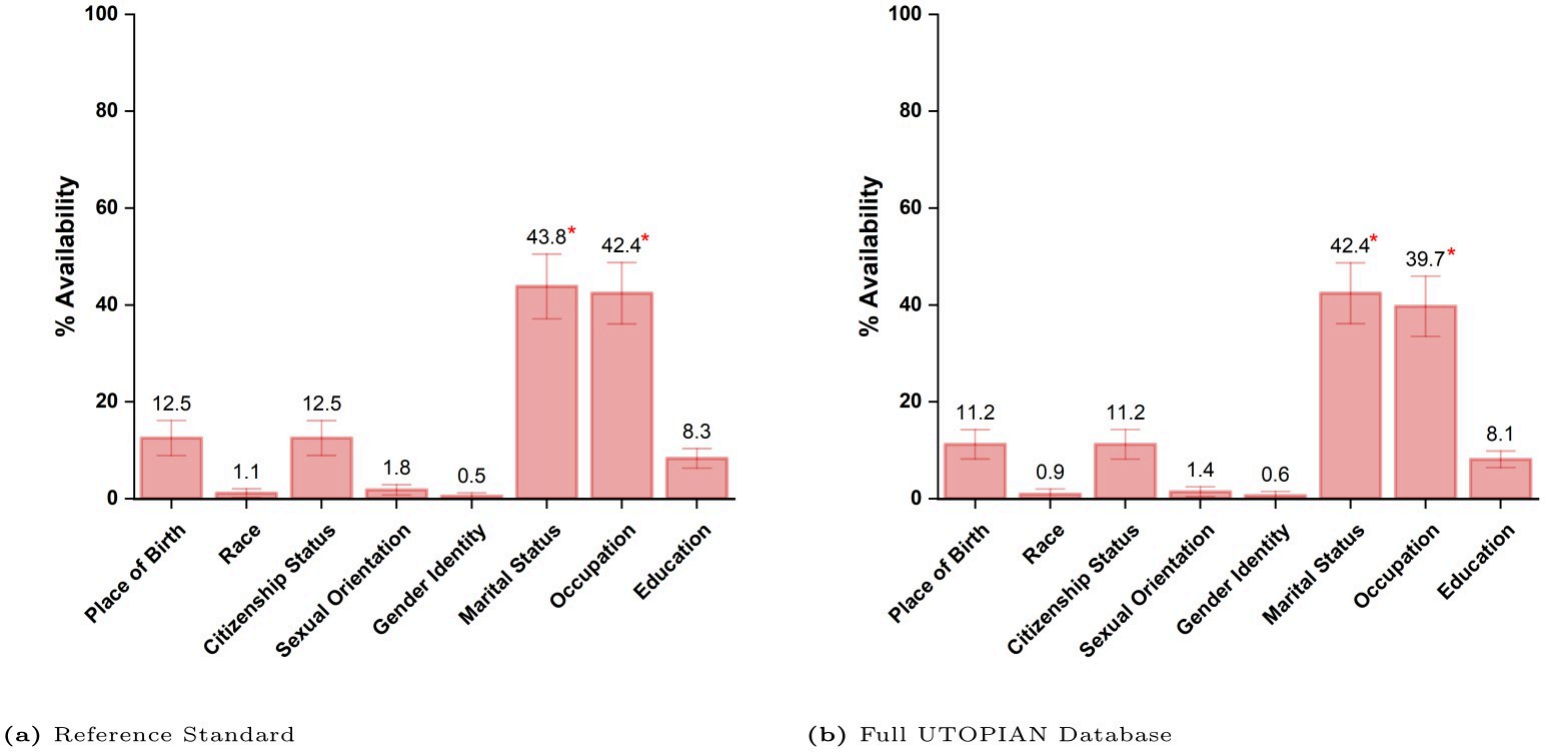
Proportion of patients with availability for each sociodemographic characteristic averaged across all clinics for both (a) the reference standard and (b) the full database. Each bar represents point estimates for each characteristic with a 95% confidence interval denoted by the error bars. The asterisk on the vertical bar denotes that the availability of data for the specific characteristic is significantly different from the other characteristics (*P* < 0.05).

Overall completeness was highest for marital status (51.0%) and occupation (47.2%) and lowest for race (1.4%), sexual orientation (2.5%), and gender identity (0.8%). A similar distribution was found in the entire database by using the machine learning model predictions. Fig 1 provides a visual representation of availability averaged across all clinics per sociodemographic characteristic for both the reference standard and the entire database. Data availability varied significantly by sociodemographic variable. Compared with marital status and occupation, the other sociodemographic variables were significantly less likely to be found in each site (*χ*^2^ > 107.5, *P* < 0.05) with an almost two-fold difference between occupation and the other characteristics (relative risk: 1.8, confidence interval [CI]: 1.7 to 1.8) and marital status and other characteristics (relative risk: 1.9, confidence interval [CI]: 1.8 to 2.0).

Table 4 summarizes the descriptive statistics for each characteristic found in the EMR. Place of birth and citizenship status had similar missingness rates (84.3% and 84.5%, respectively). However, the most frequent values for both categories did not provide detailed information about the patient’s sociodemographic status.

**Table 4:** Descriptive statistics and missingness rate for each sociodemographic characteristic within the clinical text in the EMR. For education status, whether a patient is currently studying or has completed their degree was distinguished using separate categories.

Characteristic	Summary	Descriptive Statistics (N = 4,375)
**Place of Birth**	*No. of records missing, n (%)*	3,690 (84.3)
	*No. of categories, n*	76
	*3 most frequent values, n (%)*	‘Other’: 252 (5.8)
		‘Canada’: 201 (4.6)
		‘Philippines’: 17 (0.4)
**Race**	*No. of records missing, n (%)*	4,313 (98.6)
	*No. of categories, n*	12
	*3 most frequent values, n (%)*	‘White—European’: 20 (0.5)
		‘White—North American’: 12 (0.3)
		‘Middle Eastern’: 8 (0.2)
**Citizenship Status**	*No. of records missing, n (%)*	3,699 (84.5)
	*No. of categories, n*	6
	*3 most frequent values, n (%)*	‘Immigrant’: 454 (10.4)
		‘Citizen’: 212 (4.8)
		‘Permanent Resident’: 5 (0.1)
**Sexual Orientation**	*No. of records missing, n (%)*	4,264 (97.5)
	*No. of categories, n*	5
	*3 most frequent values, n (%)*	‘Heterosexual’: 65 (1.5)
		‘Homosexual’: 40 (0.9)
		‘Bisexual’: 5 (0.1)
**Gender Identity**	*No. of records missing, n (%)*	4,341 (99.2)
	*No. of categories, n*	6
	*3 most frequent values, n (%)*	‘Cis-woman’: 16 (0.4)
		‘Cis-man’: 13 (0.3)
		‘Trans-man’: <5
**Marital Status**	*No. of records missing, n (%)*	2,143 (49.0)
	*No. of categories, n*	8
	*3 most frequent values, n (%)*	‘Married’: 1,423 (32.5)
		‘Single’: 269 (6.1)
		‘Relationship’: 188 (4.3)
**Occupation**	*No. of records missing, n (%)*	2,309 (52.8)
	*No. of categories, n*	7
	*3 most frequent values, n (%)*	‘Occupation details’: 1,220 (27.9)
		‘Employed’: 346 (7.9)
		‘Retired’: 323 (7.4)
**Education Status**	*No. of records missing, n (%)*	3,952 (90.3)
	*No. of categories, n*	14
	*3 most frequent values, n (%)*	‘University-undergraduate: 137 (3.1)
		‘University-graduate: 74 (1.7)
		‘In-undergraduate’: 52 (1.2)

### 3.4 Relationship between EMR vendor and completeness rates

**Fig 2:**
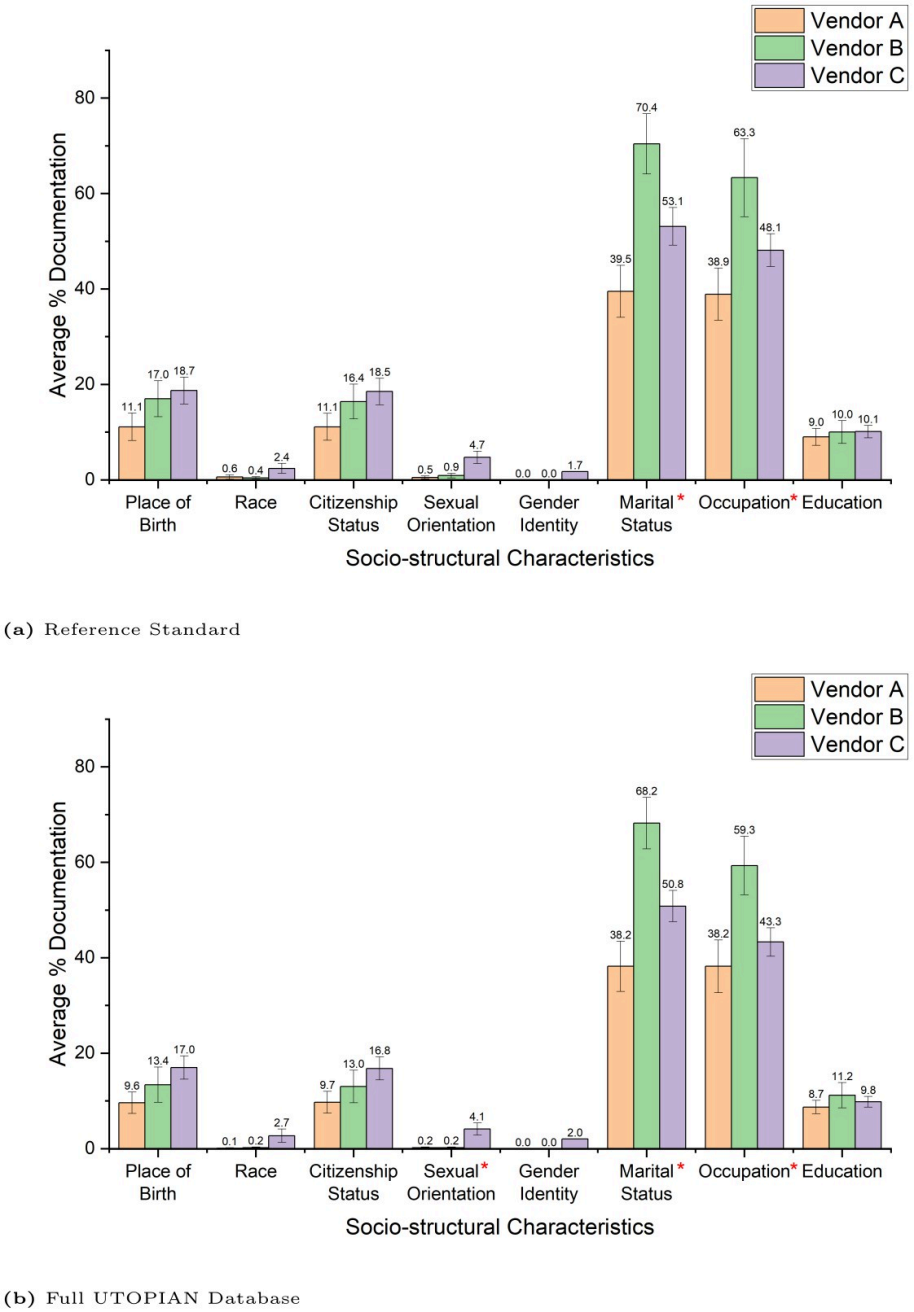
Average percent of documentation rates for each sociodemographic characteristic based on vendor for the reference standard (a) and the full UTOPIAN database (b). The error bars were calculated based on the standard error for each characteristic’s documentation rates. The asterisk on a characteristic denotes that documentation rates are statistically different across vendors (*P* < 0.05).

Fig 2 provides an overview of the average availability of each characteristic in the EMR by vendor type for the reference standard and the full database. The results were statistically significant for marital status and occupation in the reference standard and for sexual orientation, marital status, and occupation in the full database (*χ*^2^ > 6.0, *P* < 0.05). Vendor A had the lowest documentation rates (with the exception of race) while Vendor C had the highest documentation rates for all characteristics except for marital status and occupation. Vendor B significantly had the highest documentation rates for both occupation and marital status.

### 3.5 Relationship between clinic and completeness rates

**Fig 3:**
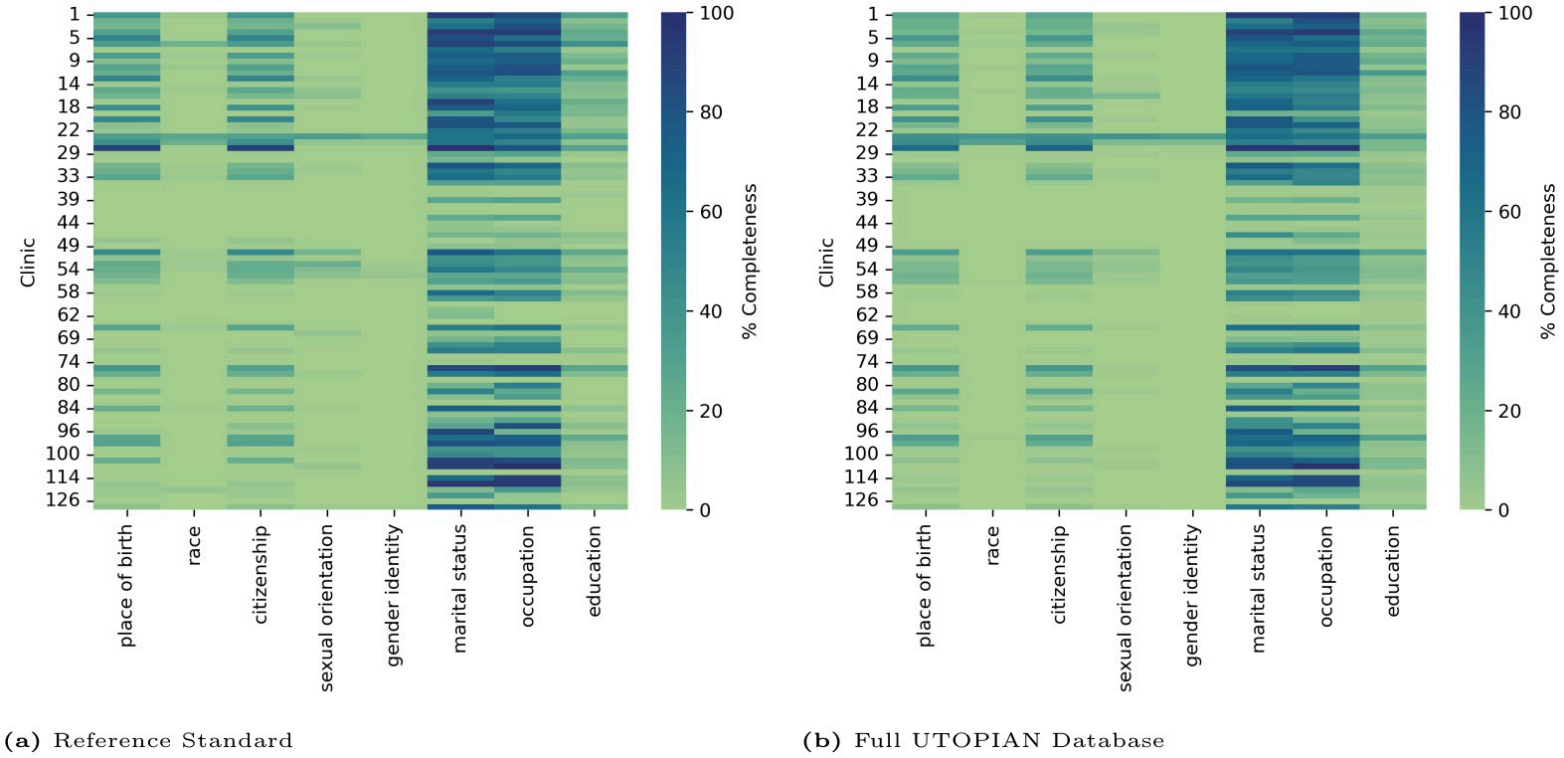
Summary of completeness of sociodemographic characteristics by clinic for (a) the reference standard and (b) the full database.

There was significant variability in terms of the completeness rates of most characteristics at the clinic level in which the highest variation was in the documentation of occupation (median: 47.2, interquartile range: 60.6%) and marital status (median: 45.6, interquartile range: 59.7%). There was a uniform lack of documentation across all clinics for race, sexual orientation, and gender identity which exhibited little to no variation in documentation rates across clinics. Fig 3 is a heatmap of the completeness rates for each sociodemographic characteristic per clinic across the reference standard and the full database. Table 5 provides the median completeness rates of each sociodemographic characteristic across all clinics in the reference standard and full database and the clinic data’s first and third quartile.

**Table 5:** Median and distribution of completeness rates presented in terms of the first and third quartiles for sociodemographic characteristics across the clinics for the reference standard and the full database.

Completeness Rate						
Characteristic	Median (%)		1st quartile (25 to 56%) (%)		3rd quartile (66 to 75%) (%)	
	Sample	Full Database	Sample	Full Database	Sample	Full Database
**Place of Birth**	2.5	3.5	0.0	0.1	21.4	21.5
**Race**	0.0	0.04	0.0	0.0	0.0	0.2
**Citizenship Status**	3.0	3.2	0.0	0.1	21.4	21.2
**Sexual Orientation**	0.0	0.1	0.0	0.0	0.9	0.6
**Gender Identity**	0.0	0.0	0.0	0.0	0.0	0.0
**Marital Status**	45.6	48.7	11.9	8.0	71.6	68.6
**Occupation**	47.2	42.0	7.9	10.8	68.5	61.2
**Education**	5.9	6.4	0.0	1.4	11.7	10.8

### 3.6 Relationship between physician and clinic variables and completeness rates

**Fig 4:**
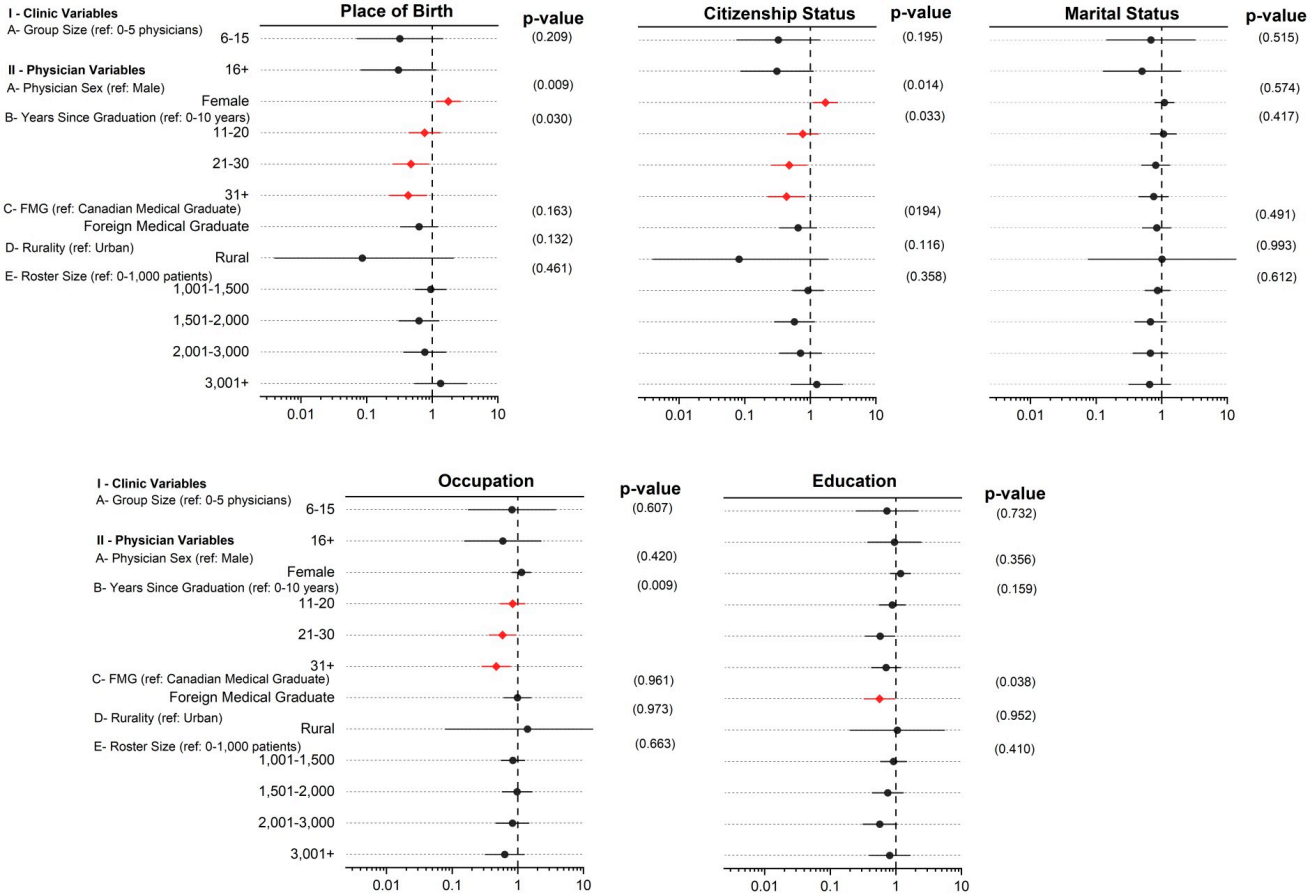
The effect of various physician and clinic variables on completeness rates for sociodemographic characteristics. Foreign vs Canadian medical graduate is abbreviated here as FMG. The variables that have a statistically significant effect on completeness rates are highlighted in red with a diamond shape. Error bars represent the 95% confidence interval. The dotted line at 1 indicates no association between the two variables. The p-value represents the statistical significance of the results. The reference category for each variable is listed in between brackets.

We found three physician characteristics (physician sex, years since graduation, and foreign vs Canadian medical graduate) had statistically significant effects on documentation rates of sociodemographic characteristics. The model was unable to converge for race, sexual orientation, and gender identity due to the extreme imbalance in class distribution.Fig 4 provides a visual representation of the association between the different physician and clinic variables and the documentation rates of the sociodemographic characteristics.

## 4 Discussion

This is the first study, to our knowledge, reporting on the quality of documentation of a wide range of sociodemographic characteristics in an Ontario EMR repository. Overall, our findings suggest that documentation varied significantly between characteristics where most were poorly documented. Marital and occupation information were the only two variables that had substantial documentation rates in which we found an almost two-fold difference between the availability of this information compared to the other variables. Race, sexual orientation, and gender identity continued to be poorly documented across all clinics with a more than 97.5% missingness rate. Furthermore, we found poor documentation of the employment status of patients as most data contained information on the details of the job rather than the status of work. Citizenship status was rarely documented but mainly assumed based on birth location. We also found variations in documentation rates across clinics and EMR vendors with the highest variation being in the documentation of occupational information followed by marital status. Finally, we found a few physician variables that significantly influence documentation rates. These findings corroborate previous studies on EMR quality assessment [23, 26, 27] and highlight how information loss results from various factors at both organizational and physician levels, particularly where consistent data collection practices for crucial sociodemographic factors are lacking.

A similar distribution of completion rates for all sociodemographic characteristics was found in the full UTOPIAN database based on the results of our best-performing machine learning algorithm. Furthermore, we found that sexual orientation varied by EMR vendor across the full UTOPIAN database where such variation was not found in the reference standard. Clinic variability across the full UTOPIAN database showed slightly less variation for all sociodemographic characteristics (except for race and marital status) when compared to the reference standard. Of note, there was a 10.2% decrease in the interquartile range for occupation in the full UTOPIAN database compared to the reference standard. Therefore, the full database may have slightly more consistent documentation of occupation across clinics than the estimated results based on the reference standard. Our results show that such tools can provide a reference standard for future studies on the prediction of EMR completeness rates for sociodemographic factors. However, there are some limitations to this work, especially on a data-driven level. For example, the machine learning models were unable to optimize for the classification of sexual orientation due to an inadequate training sample. Therefore, there is only so much improvement that can be made on a technical level without adequate data collection which is why we emphasize the need for better data standardization.

We emphasize that the collection of more distinctive data and standardizing race information at the point of service could lead to better characterization of individuals and include local differentiation in terminology [34]. Various research has found an association between health and racial background [35–41]. Furthermore, institutionalized racial and internalized discrimination plays a role in patient’s health outcomes and health service use [42]. The collection of this data could lead to potential advances in health research [24, 42, 43]. We found that race was the second least documented sociodemographic characteristic in the EMRs. Without standardization and increasing documentation of this means of measurement, it will remain abundantly difficult to compare health-related data based on these constructs. This can be noted by the lack of documentation of race data across clinics and EMR vendors where little variability was found but rather a consistent lack of information collection in all EMR vendors and clinics involved.

The list of sexual orientation and gender identity values included a wide array of identities and sexual preferences that patients may possess. However, our findings show that for sexual orientation only heterosexuality and homosexuality were somewhat documented while other values had less than 0.2% of the data provided. Approximately 4% of the Canadian population aged 15 years or older as of April 5^th^, 2018 identify as being a part of the LGBTQ2+ community where 0.33% of the population are transgender or non-binary [44]. With a 2.5% likelihood of finding sexual orientation information and a staggering low documentation rate of 0.8% for gender identity, it is evident that these variables (as well as race) require the most attention in future data collection projects. Of note, one of the EMR vendors recently updated their software to include the collection of pronouns. However, providing the necessary tools (distinct fields) to prompt documentation of these characteristics may not be enough. Rather, attention needs to be provided to understand the lack of documentation of these characteristics.

Despite marital status and occupation data being more available in the EMR, there are still some improvements that must be made in data collection. Completeness of marital status and occupational information showed the highest variation across clinics and vendors with the least amount of distinct information provided on employment status compared to all other characteristics. These findings suggest that there is a need to promote higher documentation rates of these characteristics to ensure that we can provide evaluations of the impact of sociodemographic information on health and healthcare. This is especially important in a Canadian setting as marital status was shown to affect health-related quality of life more in Canada than in the US population [35]. Furthermore, occupation and education also play an important role in patients’ health with lower income and educational attainment decreasing health quality [38]. Occupational information requires the most standardization protocols to be implemented so more accurate data can be extracted from the EMR.

EMR vendor documentation variability can be influenced by factors such as the availability of standard phrases and paragraphs, discrete variables or templates, and automatic object insertion (eg. bringing in clinical values from various parts of the EMR). Such tools can significantly enhance information collection. Only Vendor C, which had the highest documentation rates for the majority of characteristics, had a more structured template that contained boxes with discrete variables for documentation of various sociodemographic characteristics. However, the discrete variables provided are for documenting occupation, marital status, and education information and the availability of these characteristics did not reflect any improvement when compared to the other two vendors. A previous study examined the activation of an EMR-based social determinants of health screening tool and found significant variation in its documentation across a national network of community health centers, indicating challenges in achieving widespread adoption [45]. Therefore, any change in EMR structure may not directly affect documentation rates if guidance to healthcare organizations on how to conduct social determinants of health screening using the EMR-based tools is not provided [46]. Based on our findings, only two clinics in Vendor B, which significantly had the highest documentation rates for marital status and occupation, provided the majority of this information. However, it is essential to have a balance between structure in the EMR and excessive data entry requirements since the latter can result in physician stress and burnout [47]. Our findings showcased this as Vendor A had the lowest documentation rates and the least user-friendly EMR structure for entering this type of information, resulting in a more time-inducing documentation process during the already short primary care visits. Therefore, vendors must ensure that EMR systems provide the needed functionality for easy reporting and use to ensure that clinics have the necessary tools to adequately document sociodemographic factors. EMRs can transform healthcare if the systems are appropriately designed and the data captured is accurate [48]. However, little has been done yet for the widespread implementation of EMR usability guidelines that have been published in the literature over the past few decades.

On the other hand, the variability between clinics in documentation rates may show that some clinics may place more emphasis on supporting social care or have purposely made policies or procedures for documenting these characteristics. Practice-level interventions and more general system changes are necessary steps to promoting more equitable healthcare services [49]. For example, including a universal screening protocol and providing more incentives to screen for sociodemographic factors can enhance documentation practices [50]. Such changes can fortify the social accountability mandate of family physicians.

Our measures of real-world sociodemographic data showed variation in documentation based on physician and clinic variables. We found three out of the six studied physician and clinic variables were significantly associated with the availability of patient sociodemographic data in their charts. Physician sex affected the documentation rates of place of birth and citizenship status where female physicians were more likely to document these two characteristics. The number of years since a physician graduated influenced the availability of place of birth, citizenship status, and occupation in the EMR in which physicians who graduated 0-10 years before the data extraction date were more likely to document these characteristics. Finally, whether a physician graduated from a foreign vs a Canadian medical university influenced how likely they were to document patients’ education status with a higher likelihood of documentation found for Canadian medical graduates. Therefore, increasing documentation of patients’ sociodemographic factors in healthcare must be facilitated from the top down, starting at the healthcare system level to the physician level, for the universal implementation of documentation practices [50].

Improvement in the quality of documentation of sociodemographic factors in the EMR depends on the consistent and complete entry of data by all participants involved in patient care [51]. However, in practice, this might be difficult to sustain over time, especially with staff turnover. Furthermore, the collection of this information also requires participation from patients. Physicians may perceive patient reluctance to disclose this information as an interpersonal barrier for assessing sociodemographic factors [50]. Martial status and occupation may be easier to disclose by patients and inquire about by medical providers, hence the higher documentation rates. Race, sexual orientation, and gender identity are more sensitive topics that patients may not feel comfortable disclosing this information to providers [19, 52–54]. Furthermore, some patients and/or physicians may feel discomfort for fear of bias in their medical care towards recording sociodemographic information in the cumulative patient profile which tends to sit at the forefront of EMR records and automatically become included in referrals to specialists. Therefore, methods to mitigate such challenges need to be made to increase rates of documentation of sociodemographic information in a medical setting as such information is crucial in increasing health equity.

A previous study that assessed the quality of primary care EMR data in Alberta found that sociodemographic factors such as ethnicity, occupation, and education were largely incomplete and highly variable [26]. For example, they found over 3500 unique entries for occupation and more than 75 distinct entries for ethnicity. Ethnicity was missing for 95.8% males and 95.6% females, occupation was missing for 71.6% males and 74.0% females, and education had a missingness rate of 97.4% for males and 97.5% for females. Previous studies have identified several barriers for the collection of sociodemographic data including a lack of agreement on which questions to ask, how the questions should be worded, the best approach to survey patients, and general concerns over the disruption of the therapeutic relationship if such questions are asked [55, 56]. One study assessed the feasibility of using a self-administered survey linked to EMRs in a family medicine clinic in Toronto for capturing sociodemographic information [57]. This included information on patients’ place of birth, immigration status, race, gender, and sexual orientation. They found that the rate of valid responses for each question was high, ranging from 84% to 100%, showcasing that this data collection tool is feasible and acceptable for enhancing the capture of sociodemographic information in primary care EMR data.

There are several limitations in our work. First, some variables contain assumptions of the information provided in the clinical text which cannot be used as a clear indication of documentation. This may have slightly exaggerated the documentation rates of certain variables. However, the number of assumptions made for all variables (excluding citizenship status) was minuscule with less than 10 labeled entries containing any assumed information. Second, our data contains information on patients from a large multi-ethnic diverse urban center with an under-representation of rural areas of the province. Therefore, a key limitation of our study concerns its generalizability. However, the healthcare providers that contributed data to this study serve a well-populated and diverse metropolitan area resulting in a study sample with high variability in sociodemographics. Third, our documentation rates were limited to information recorded in the semi-structured cumulative patient profiles. However, sociodemographic data could be buried in the free text fields of clinical notes as physicians may tend to document this information in other areas of the EMR [58]. Finally, this study is limited by its focus on the completeness of sociodemographic data at a single point in time, using the most up-to-date status for each sociodemographic factor in all our analyses. Therefore, we did not account for potential changes in these data over time or the timing and frequency of data entries. Future research should explore the dynamics of sociodemographic data in EMRs, including how often these data are updated and the impact of such changes on clinical decision-making.

A key benefit of the widespread use of EMRs was their ability to improve upon the quality of medical data and their usefulness in research prospects. However, high-quality and complete data entry in EMRs is essential to use the data for reliable primary care measures and health outcomes [59]. In fact, rather than leading to improvements in the quality of data, the introduction of EMRs has resulted in the documentation of a larger quantity of bad data [48]. The observed range of availability of sociodemographic variables in our data assessment suggests that there is a critical need for creating more user-friendly EMR structures for documenting sociodemographic information, encouraging healthcare workers to document this information in EMRs, and standardization of the content to support the use of this information in clinical care and research settings. These findings address local challenges in Ontario, Canada, but are also likely reflective of broader, global trends in EMR data management. The insights underscore the importance of adopting universal best practices for sociodemographic data documentation, which could apply to healthcare systems worldwide. Our study highlights the need for international collaboration and standardization in EMR systems to ensure that sociodemographic data is consistently and accurately captured across different contexts.

The current standards for completeness rates on these characteristics provide us with limited and poorly represented data that cannot be used to assess the role these factors play in health. Although it may be unrealistic to aim for full completeness of these data elements, it is reasonable to assume that the current data collection standards can be improved upon at the point of care. Various strategies have shown improvement in completeness rates of EMR data such as utilizing an allocated data entry clerk [25], providing feedback reports to clinicians on the quality of the data [60], or more intensive techniques such as mandated national EMR standards [61]. Although such data standardization schemes exist, data quality issues remain permanent for sociodemographic information collection.

## 5 Conclusion

Sociodemographic information can provide crucial information on health outcomes. However, the completeness of data documentation has persistently proven to be poor with large variations in completeness at the physician, clinic, and vendor levels. This study examined the current practices employed for collecting this information at the point of care. We found that the completeness of sociodemographic data in EMRs requires substantial improvement before this data can be reliably used for secondary purposes. The lack of sociodemographic information in patient health records is multipronged and could be caused by insufficient availability of standardized specific variables, inadequate documentation standards, and potentially even lack of inquiry of such information by healthcare providers and logging of such information in clinical notes rather than the more accessible cumulative patient profile due to privacy concerns. Emphasis on the importance of sociodemographic information on health outcomes needs to be made to obtain more useful and diverse data. Further research is needed to provide meaningful intervention schemes.
